# Incidence, Clinico-Radiological Features and Outcome of Skull Base versus Non-Skull Base Meningiomas Treated in Kuala Lumpur General Hospital: A Five-Year Experience

**DOI:** 10.21315/mjms2018.25.3.9

**Published:** 2018-06-28

**Authors:** Chan Chee Kong, Regunath Kandasamy, Saffari Haspani, Zamzuri Idris, Jafri Malin Abdullah

**Affiliations:** 1 Centre for Neuroscience Services and Research, Universiti Sains Malaysia, Jalan Sultanah Zainab 2, 16150 Kubang Kerian, Kelantan, Malaysia; 2 Department of Neurosciences, School of Medical Sciences, Universiti Sains Malaysia, Jalan Sultanah Zainab 2, 16150 Kubang Kerian, Kelantan, Malaysia; 3 Department of Neurosurgery, Hospital Kuala Lumpur (HKL), Jalan Pahang, 53000 Kuala Lumpur, Malaysia

**Keywords:** meningioma, skull base, outcome

## Abstract

**Background:**

Meningiomas are the most common intracranial tumours; they account for 13%–26% of all the primary intracranial tumours. Skull base meningiomas make up 25% of all meningiomas and are one of the most difficult intracranial tumours to be managed surgically. This is due to the fact that it is difficult to approach the lesions which are also close to vital structures such as cranial nerves and major blood vessels. Despite the abundance of these cases in Malaysia, local data on meningiomas is scarce.

**Methods:**

This is a retrospective study consisting of 199 patients with meningiomas who have been operated at the Kuala Lumpur General Hospital from January 2010–December 2014. They were categorised into skull base and non-skull base groups. Demography, tumour characteristics, and patient outcomes were analysed. Kaplan-Meier survival curves as well as Cox hazard univariable and multivariable regressions for the possible predictors of survival were analysed.

**Results:**

97.5% of the patients (*n* = 194) had WHO grade I meningioma and only five patients had WHO grade II meningioma. There was a female predominance (*n* = 134; 67.3%), with a male-to-female ratio of 1:2. Some 27.1 % patients had skull base meningiomas. Patients with skull base meningiomas had poorer outcomes and discharge conditions (*n* = 23; 42.6% *P* < 0.01), in addition to higher risk of incomplete resections (*n* = 34; 63% *P* < 0.01). Multivariate cox hazard regressions showed that the skull base meningioma group had four times the risk of death of the non-skull base group.

**Conclusions:**

Symptomatic meningiomas can be curative if the tumour is completely removed. Our study has revealed that skull base meningiomas which were operated locally had higher rates of incomplete resection and poorer surgical outcomes as compared to the non-skull base group. Patients with skull base meningiomas had four times the risk of death vis-à-vis non-skull base ones. More local studies are needed to look into skull base meningiomas for the improvement of its surgical outcomes.

## Introduction

Meningiomas are commonly-encountered extra-axial pathological entities that account for a significant proportion of patients seeking treatment at neurosurgical centres worldwide. In the United States, the annual incidence of these tumours is estimated to be 6.0 per 100,000 people per year. This figure surpasses that of gliomas, notwithstanding the fact that meningiomas are the most common primary tumours of the central nervous system (1). Similar epidemiological trends are seen in the Malaysian population, as per the few small case series published by regional neurosurgical centres. Authors have reported that 35% of all brain tumours that have been operated on were meningiomas (2, 3, 4). With due consideration to this epidemiological trend (which otherwise requires more data), we have retrospectively reviewed the incidence, clinical features, radiological findings, as well as outcomes of all patients with intracranial meningiomas who underwent surgical treatment at the Kuala Lumpur General Hospital over five years. This hospital is one of the largest tertiary neurosurgical referral center in our country which caters to the local population.

Meningiomas of the cranial base account for 25% of all meningiomas; the reported distribution was in the calvarial-to-skull base ratio of 2.3:1 (5). Skull base meningiomas are difficult to be managed surgically because the lesions are difficult to be approached, and that they are in close proximity to vital structures such as cranial nerves and major blood vessels (5, 6). Literature reviews have reported that skull base meningiomas are more surgically-demanding and -challenging as compared to non-skull base meningiomas (6, 7). Many countries have made skull base neurosurgery a distinct subspecialty of neurosurgery (6). Two local prospective studies on meningiomas at the General Hospital of Sarawak did not group the meningiomas into skull base and non-skull base types for comparison (2, 4). Categorisation was important for the determination of the surgical outcomes and the prevalence of meningiomas in a local neurosurgical department.

## Methodology

### Study Population

This is a retrospective review of all patients with newly-diagnosed and positive histology of intracranial meningiomas who underwent surgical treatment at Hospital Kuala Lumpur from January 2010–December 2014.

### Demography, Clinical and Radiological Characteristics

Data of interest were demography (age and gender) as well as clinical presentation. The tumours were categorised into skull base and non-skull base types, as described by Fischer and Brokinkel (5, 8–11). Skull base meningiomas include those of the olfactory groove, sphenoid ridge, tuberculum sellae, cerebellopontine angle, and petroclivus (or clivus). Non-skull base meningiomas include tumours that are found at the convexity, falx, parasagittal/supratentorial/ infratentorial areas, and at any location other than the skull base.

Evaluations of the radiological features of the patients were based on pre-operative magnetic resonance (MR) as well as computed tomography (CT) images. Tumour size and presence of oedema were determined by MR imaging. The maximum diameters of the tumours were measured and their volumes estimated using the following formula (tumour volume = ABC/2; A = maximum tumour diameter, B = diameter of the tumour perpendicular to A, and C = maximum height of the tumour) (4, 12). Hyperostosis and calcification were documented based on CT images (13).

### Surgical Extent, Complications and Tumour Histopathology

Extent of tumour resection and tumour characteristics were retrieved from the operative notes. The extent of resection–which was classified into gross total resection (GTR) and subtotal resection (STR)–corresponded with EORTC/RTOG’s definitions of Simpson 1–3 and Simpson 4 clearances respectively (14, 15). Besides, GTR was defined as “no intraoperative evidence of residual tumour with no evidence of residual tumour on post-operative contrast enhanced CT (14, 15). When either of these criteria were met, the extent of surgery was classified as subtotal (15). The occurrence of surgery-related complications were recorded. Histology of meningioma was graded as 1, 2, or 3 as per the World Health Organization’s (WHO) histopathological classification (16, 17).

### Outcome

Patient outcomes were measured with reference to the modified Rankin Scale (MRS) scores at 3 months, 6 months, 12 months, and 18 months (18). For survival analysis, overall survival (OAS) was calculated from the date of diagnosis to either the date of death or that of the last follow-up (5, 19–20).

### Statistical Analysis

Descriptive statistics were analysed using Strata version 22 and SPSS version 24. Categorical data were expressed as proportions while continuous data were presented as means ± standard deviations (SD). Differences in gender and age were assessed using α-test and *t*-test respectively. Maximum diameters of the tumours and calculated tumour volumes were represented as means ± SDs. Overall survival (OAS) was calculated from the date of diagnosis to either the date of death or that of the last follow-up. After that, Kaplan-Meier survival curves were plotted to estimate the survival rates (21). Univariate and multivariate cox regression analyses were conducted to identify the predictors of poor outcomes. The statistical significance was set at 0.05.

## Results

### Descriptive Analysis prior to Grouping

A total of 1149 intracranial brain tumour cases were operated between January 2010 and December 2014. There were 199 patients with newly-diagnosed meningioma, most of whom had WHO grade I meningioma (*n* = 194, 97.5%); the remaining five patients had WHO grade II meningioma. There were no newly-diagnosed WHO grade III meningioma patients during the study period. A female predominance (*n* = 134; 67.3%) was noted, along with a male-to-female ratio of 1:2. Most of the patients were of Malay ethnicity (*n* = 138; 69%), and the highest incidence was seen in the 40–60-year-old age group. (*n* = 124; 62.3%).

### Descriptive Analysis after Grouping

Fifty four cases (27%) were skull base meningiomas while 145 cases (73%) were non-skull base meningiomas.

### Patients’ Baseline Characteristics (Table 1)

Most of the patients in both groups were of Malay ethnicity; i.e. 39 cases (72.2%) in the skull base group versus 99 cases (68.3%) in the non-skull base group. Females predominated in the skull base group (*n* = 38, 70%). The mean age of the skull base meningioma patients at time of diagnosis was 51.7 years (SD = 11.01), approximately three years younger that of the non-skull base group (54.1 years; SD = 9.4). Both categories were most frequently seen in the 40–60-year-old age group (skull base group: *n* = 33, 61.1%; non-skull base group: *n* = 91, 62.8%).

Headache was the most common complaint, whereby 94.4% (*n* = 51) in skull base group and 91% (*n* = 132) in the non-skull base group had this symptom. The mean duration from the onset of complaints to the seeking of medical treatment in the skull base meningioma group was 4.18 months (SD = 3.4, *P* = 0.990), while the non-skull base group 4.17 months (SD = 3.05, *P* = 0.990).

#### i. Tumour characteristics (Table 2)

The majority of skull base meningiomas (*n* = 31; 57%) and non-skull base meningiomas (*n* = 95; 65%) were present in the right cerebral hemisphere. The mean tumour diameter of the skull base meningiomas was 4.76 cm (SD = 1.17) while that of the non-skull base meningiomas was 4.79 cm (SD = 1.37). Pre-operatively, the mean tumour volume of the non-skull base meningiomas and skull base meningiomas were similar 51.4 cm^3^. Post-surgery, the mean tumour volumes were 0.707 cm^3^ (SD = 3.08) and 5.278 cm^3^ (SD = 6.65) for the non-skull base and skull base meningiomas respectively; with significant *P*-value of < 0.001.

Oedema was present in 94.4% (*n* = 51) of skull base meningioma cases and 89.7% (*n* = 130) of non-skull base meningioma patients. In the skull-base meningioma group, 30 cases (55.6%) had radiological evidence of hyperostosis, while 16 patients (29.6%) had calcification.

Skull base meningiomas had a mean duration of surgery of 7.68 h (SD = 1.21), which was longer than the 4.4 h (SD = 1.01) for non-skull base meningioma (*P* < 0.001). Some 87.6% of the non-skull base meningiomas (*n* = 134) were completely resected, while 76.1% of the skull base meningiomas (*n* = 35) were incompletely resected.

#### ii. Characteristics of outcomes (Table 3)

Patients in the skull base group had a significantly longer mean duration of ICU stay (2.68 days, SD = 2.6) as compared to those in the non-skull base group (1.1 days, SD = 0.93) (*P* < 0.001). Meanwhile, the skull base group also had a significantly longer mean duration of hospital stay (11.8 days, SD = 6.9) as compared to the non-skull base group (7.12 days, SD = 4.1) (*P* < 0.05).

Post-surgery, 10 patients (18.5%) in the skull base group died. Also, four patients were fully-dependent and nine patients were partially-dependent in their activities of daily living post-surgery. Some 89.6% of the patients in the non-skull base group and 53.7% of those in the skull base group (*n* = 29) had uncomplicated surgeries. Twenty one patients in the skull base group (39%) required re-surgery. The indications included haematomas (*n* = 10), infarcts (*n* = 3), and infected wounds (*n* = 8).

#### iii. Functional outcomes (Table 3)

The patients were followed-up and assessed three months, six months, 12 months, and 18 months post-surgery. Surveillance MRI brain reports were reviewed and documented. The functional outcomes were categorised as mildly disabled [Modified Rankin Scale (MRS) 0–2], moderately disabled (MRS 3–4), severely disabled (MRS 5), and death (MRS 6). At the 3-month follow-up, 93.8% of the non-skull base meningioma patients were mildly disabled (*n* = 136) while 68.5% of the skull base meningioma patients (*n* = 37) were mildly disabled.

At the 12-month follow-up, 94.5% of those in the non-skull base group were mildly disabled; one patient in the moderately disabled category improved into the mildly disabled category. By 18 months, two more patients in the skull base group died due to other reasons; resulting in a total of 12 post-operative deaths in the skull base group.

### Outcomes and Overall Survival until Final Follow-Up Session

**Figure 1:**
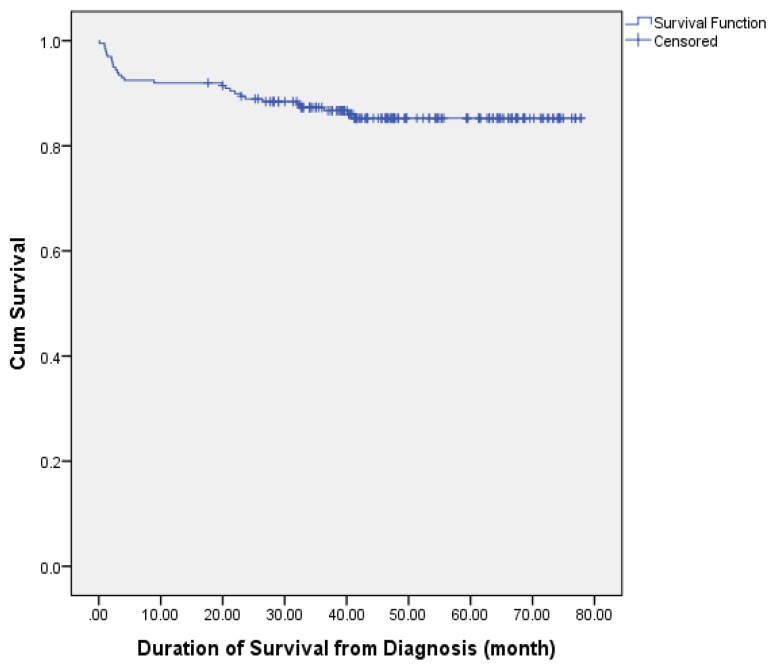
Kaplan Meier survival curve for meningioma operated in General Hospital Kuala Lumpur

The follow-up durations and overall survival (OAS) of the 199 intracranial meningioma patients were calculated from the date of diagnosis to either the date of death or that of the last follow-up. Our study revealed a median survival of 37.6 months (SD = 20.94) for the skull base group as compared to 47.8 months (SD = 18.2) in the non-skull base group (*P* = 0.187) (Table 3). As per the Kaplan-Meier analysis the survival rates at 12 months, 24 months, and 80 months were 90%, 87%, and 85%, respectively (Figure 1).

### Predictors of Survival as per Univariate and Multivariate Cox Regressions (Tables 4, 4a and 4b)

There were 18 deaths among the meningioma patients throughout the study period. Univariate and multivariate Cox hazard regressions were calculated based on the patients’ outcomes. After adjustments for the potential confounding variables in a multivariate model (Table 4), significant differences were noted between the location of meningioma and the patient outcomes (skull base versus non-skull base adjusted hazard ratio (HR) = 4.27; 95% confidence interval (CI) = 1.504–12.129; *P* = 0.006 and WHO grade (Grade II versus Grade I) adjusted HR = 5.22; CI = 1.256–21.673; *P* = 0.023. Gender, ethnicity, comorbidities, age group, and extent of meningioma resection did not have significant effects on patient outcomes (Table 4).

The possible confounding variables were regrouping (Table 5) and the Cox regression that was adjusted to gender, comorbidities, WHO grading, and extent of meningioma resection revealed that skull base meningioma patients had 4 times the risk of death of the non-skull base meningioma patients (adjusted HR = 4.22; 95% CI = 1.53–11.66; *P* = 0.005).

## Discussion

This study has identified the characteristics of newly-diagnosed and histologically-confirmed meningiomas. However, the small number of WHO grade II (*n* = 5) meningiomas and absence of WHO grade III meningiomas led to the inability to compare the meningiomas based on WHO grading. The grouping of meningiomas based on location (skull base versus non-skull base) therefore allowed the delineation for the purpose of comparing and exploring the incidence and surgical outcomes of these patients.

WHO grade I meningioma was far more common (*n* = 194; 97.5%) as compared to WHO grade II, and this was comparable to other studies (5, 8). Besides, out of the 199 patients, females predominated (*n* = 134; 67.3%) over males (*n* = 65; 32.7%) with a ratio of 2:1. This was also comparable with other studies (5, 8, 22). Most of the patients were of Malay ethnicity (*n* = 138; 69.3%); this was reflective of the local demography.

Skull base meningiomas (*n* = 54) constituted 27.1% of all intracranial meningiomas, the majority of which were WHO grade I (*n* = 52, 96.3%). Seventy percent of the skull base meningiomas occurred in females (*n* = 38) as well. Skull base meningiomas tended to be diagnosed earlier at a mean age of 51.7 years (SD = 11.01) as compared to the mean age of 54.1 years (SD = 9.4) in the non-skull base group.

Both categories had the highest prevalence in 40–60-year-olds (skull base group: *n* = 33, 61.1%; non-skull base group: *n* =91, 62.8%), which was similar with the results of other studies (1). Fifty-one patients with skull base meningiomas (94.4%) had radiological evidence of oedema; 30 cases (55.6%) had evidence of hyperostosis and 16 patients (29.6%) had calcification.

Skull base meningiomas were a predictor of poorer outcomes (*n* = 23; 42.6% *P* < 0.01) and incomplete resections (*n* = 34; 63% *P* < 0.01). The current study revealed that 75% of all incompletely-resected meningiomas and 56.1% of poor-outcome cases in this cohort were from the skull base group. Our study reported a shorter median survival of 37.6 months (SD = 20.94) in the skull base group as compared to 47.8 months (SD = 18.2) in the non-skull base group (*P* = 0.187). Skull base meningioma patients had four times the risk for death of non-skull base meningioma cases (adjusted HR = 4.22; 95% CI = 1.53–11.66; *P* = 0.005), when adjusted for gender, comorbidities, WHO grading, and extent of resection.

The extent of resection has been established many years ago as the primary prognostic factor of recurrence (1, 9, 25). Gross total resection (GTR) was the accepted standard of care for benign meningiomas (25, 26). Simpson grade I removal of skull base meningiomas was associated with only 4% to 15% risk of long-term recurrence (7). Our study found that only 19 cases (35%) achieved total resection in the skull base group as compared to 134 cases (87%) in the non-skull base group. Doubts and worries of inducing significant morbidity and neurological sequelae in skull base meningioma surgeries could have been the reason for the abovementioned findings, as stated by Adachi et al. (23).

GTR is difficult to be done at the skull base due to the presence of important surrounding anatomical structures; aggressive resection can lead to severe morbidity (24, 25). Subtotal resection (STR), or partial resection, is performed to preserve neurological function (23, 26). Adachi et al. demonstrated the negative effect of extensive resections on the neurological outcomes of a subgroup of patients (23). Scheitzach et al. have reported that careful and complete decompression of the adjacent structures was sufficient to relieve the clinical symptoms, rather than attempting to radically resect the entire tumour shell (18, 23).

Our study has demonstrated that skull base meningiomas had a longer mean duration of surgery (7.68 h; SD = 1.21) as compared to non-skull base meningiomas (4.4 h; SD = 1.01) (*P* < 0.001). Four patients (7%) in the skull base group developed deep vein thrombosis as compared to only one patient (0.6%) in the non-skull base group. These could be attributed to the longer duration of surgery in the former. Despite requiring a longer duration of surgery, the majority of skull base meningiomas (76.1%, *n* = 35) were incompletely resected, and 38.9% (*n* = 21) required re-surgery for indications such as haematomas (*n* = 10), infarcts (*n* = 3), and infected surgical wounds (*n* = 8).

Comparatively, patients with skull base meningiomas had significantly longer duration of intensive care unit (ICU) admission, with a mean duration of 2.7 days (SD = 2.6) as compared 1.1 days (SD = 0.93) in the non-skull base group (*P* = 0.001). Meanwhile, the skull base group also had a significantly longer duration of hospital stay, with a mean duration of 11.8 days (SD = 6.9) as compared to 7.12 days (SD = 4.1) in the non-skull base group (*P* = 0.001). Cost-effectiveness in the management of skull base meningiomas as compared to non-skull base ones should never be neglected.

In this study, 18.5% (*n* = 10) skull base meningioma patients died within the post-operative period; this mortality rate was higher than that of Chen et al. (7). Two skull base meningioma patients died due to pneumonia and septicaemia secondary to urinary tract, respectively. This was due to their prolonged immobilisation after surgery. Only 57.4% (31 out of 54) of skull base meningioma patients were discharged well, as compared to 88.3% (128 out of 154 patients) of non-skull base meningioma cases.

In the case series of 73 sphenoid ridge meningiomas by Honig et al. (27), the rates of perioperative morbidity and mortality were 7% and 3%, respectively. In another case series of 117 foramen magnum meningiomas by Wu et al. (28), the perioperative mortality was 1.8%. Other studies—including Nakamura et al. (29, 30) and Spektor et al. (31)—have reported gross total resections in approximately 90% of olfactory groove and tuberculum sellae meningiomas, with a perioperative mortality of 2.8% for both (5, 7). Table 6 (Chen et al.) gives an overview of the post-surgical morbidity and mortality for skull base meningiomas; these were mainly based on date from reports published from 2000–2010 (5, 7).

The management of skull base meningiomas is challenging, even for experienced neurosurgeons (5). Our study reported that despite the longer surgery time, there was a higher rate of incomplete resection that was associated with higher morbidity and mortality rates (four times higher risk of death) in the skull base group. An increase in skull base neurosurgical training helps reduce the variability in the surgeons’ competencies. Besides, there is a dire need to improve the local technology, including the use of adjuvant radiosurgeries or even targeted molecular therapies, for skull base meningiomas (5).

## Limitations

A limitation of this study was its retrospective nature, which could have led to missing data and selection bias. Single-centered data could also have led to selection bias and hence, an untrue representation of the incidence in the local population. The small number of patients with WHO grade II meningioma was statistically insufficient to measure the relative impact of some variables on patients with grade II meningiomas.

Kuala Lumpur General Hospital still practices manual writing of cases. Entries were made by the admitting and managing medical officers, whereby clinical improvement and even factors that influenced the patients’ outcomes could have been missed. Despite the fact that the Karnofsky performance scale (KPS) is a widely-used standardised scale for measuring the ability of brain tumour patients to perform ordinary tasks (18, 24, 26), it was not utilised by the attending doctors during the study. Similarly, while the mini-mental state examination (MMSE) is commonly used to predict brain damage after meningioma surgery, its infrequent usage in this hospital has resulted in its non-inclusion in this study.

The planimetry method with thick- or thin-sliced MRI brain, as suggested by Ishi et al., was not performed to assess the post-surgical tumour volumes, so we were unable to include any such results in the study (12). The inability to obtain post-operative MRI at standardised intervals, apart from the lack of systematic volume calculations in the reports, could have led to some degree of inaccuracy of calculated extents of resection and volumes. Besides, inadequate and inconsistent MRI or other relevant radiological reports on the characteristic features of meningiomas–such as vascular signal voids, degree of peritumoural oedema, signal grading of tumour on MRI (i.e. hypo-, iso-, and hyper-signals in both T1W and T2W images), and dural tail signs (Ben Nsir et al.)–have made it difficult to analyse the radiological features in detail (13).

The majority of histopathological reports were brief; they included only WHO grading and type, immunohistological markers (such as S100 and fibronectin), as well as cell surface receptors (e.g. Ki67 and HH3) in higher-grade meningiomas (15, 16) have been inadequately reported, hence it was difficult to perform detailed histopahological analyses.

## Conclusion

In conclusion, our study has shown that meningiomas are common at the skull base; these constitute 27.1% of all intracranial meningiomas at the main neurosurgical referral center in Malaysia. The management of skull base meningiomas are more time-consuming, apart from being associated with more severe morbidities and longer hospital stays. Besides, patients with skull base meningiomas are four times likelier to die as compared to the non-skull base group. We strongly recommend that multicentric prospective studies be carried out to provide key insights not only in terms of the overall prevalence and radiological evaluations of meningiomas in Malaysia, but also the use of adjuvant radiosurgeries or even targeted molecular therapies. Also, the effects of these possible confounding variables on the clinical outcomes in patients with skull base meningiomas can be determined by such studies.

## Figures and Tables

**Table 1:** Meningiomas patients characterised by location–skull base or non-skull base (*N* = 199)

	Non-skull base (*n* = 145)		Skull base (*n* = 54)		*P*-value^a^
	
	*n*	%	*n*	%	
Age (mean, SD)	54.1 (9.4)		51.68 (11.01)		0.050^b^
Age group					
< 40	11	7.6	7	13	0.481
40–60	91	62.8	33	61.1	
> 60	43	29.7	14	25.9	
Ethnicity					
Malay	99	68.3	39	72.2	0.022^c^
Chinese	29	20	8	14.8	
Indian	15	10.3	2	8.5	
Others	2	1.4	5	3.5	
Gender					
Male	49	33.8	16	29.6	0.578
Female	96	66.2	38	70.3	
Comorbidities					
No	73	50.3	27	50	0.233
Single	49	33.8	23	42.6	
Diabetes	39	26.9	20	37	0.556^c^
Hypertension	9	6.2	3	5.6	
IHD	1	0.7	0	0	
Multiple	23	15.9	4	7.4	
Co-morbids on medical treatment	70	97.2	27	100	0.817^c^
Compliance to medical treatment	34	47.2	16	59.3	0.564
Complaints:					
Headache	132	91	51	94.4	0.014^c^
Headache alone	13	8.9	1	1.8	0.141^c^
Headache plus others	119	82.1	50	92.6	0.141^c^
Headache + Nausea Vomiting	69	47.6	30	55.6	< 0.001^c^
Headache + Weakness	26	17.9	0	0	
Headache + Fits	23	15.9	3	5.5	
Headache + Visual Disturbance	1	0.7	17	31.5	
Others without headache	13	8.9	3	5.6	0.141^c^
Visual disturbance alone	0	0	2	3.7	< 0.001^c^
Weakness alone	11	7.6	0	0	
Fits alone	2	1.3	0	0	
Hearing problems alone	0	0	1	1.9	
Duration of symptoms prior to seeking treatment (means, SD)	4.17 (3.05)		4.18 (3.4)		0.990^b^

a Chi-square test

b Independent *t*-test

c Fisher exact test

**Table 2:** Tumours characterised by location–skull base or non-skull base (*N* = 199)

	Non-skull base (*n* = 145)		Skull base (*n* = 54)		*P*-value^a^
	
	*n*	%	*n*	%	
Hemisphere					
Right	95	65.5	31	57.4	0.291
Left	50	34.5	23	42.6	0.291
Tumour Size (cm)/volume (cm^3^) d					
Largest diameter (mean, SD)	4.79 (1.368)		4.76 (1.17)		0.430^b^
Pre-surgery volume (mean, SD)	51.4 (19.15)		51.4 (19.4)		0.989^b^
Post-surgery volume (mean, SD)	0.707 (3.08)		5.278 (6.65)		< 0.001^b^
Radiological features^e^					
Presence of calcification	39	26.9	16	29.6	0.701
Presence of oedema	130	89.7	51	94.4	0.295
Presence of hyperotosis	90	62.1	30	55.6	0.404
WHO grading					
Grade I	142	98	52	96.3	0.614^c^
Grade 2	3	2	2	3.7	0.614^c^
Duration of surgery in hours (mean, SD)	4.4 (1.01)		7.68 (1.21)		< 0.001^b^
Simpsons grading					
1	96	66.2	2	3.7	< 0.001
2	27	18.6	7	13	
3	11	7.59	10	18.5	
4	11	7.59	35	64.8	
Complete resection (Simpsons 1–3) f	134	87.6	19	12.4	< 0.001
Incomplete resection (Simpsons 4) f	11	23.9	35	76.1	< 0.001

a Chi-square test

b Independent *t*-test

c Fisher Exact test

d Volume calculation as quoted in Ishi et al. (12)

e radiological feature as quoted in Ben Nsir et al. (13)

f Simpson grade of resection and corresponding EORTC/RTOG definitions of extent of resection as quoted in Jenkinson et al. (14)

**Table 3:** Outcome of operated meningioma patients based on location in GHKL (*N* = 199)

	Non-skull base (*n* = 145)		Skull base (*n* = 54)		*P*-value^a^
	*n*	%	*n*	%	
Duration of ICU admission (mean, SD)	1.1 (0.93)		2.7 (2.6)		< 0.001^b^
Duration of Hospital stay (mean, SD)	7.12 (4.1)		11.8 (6.9)		< 0.001^b^
Discharge condition					
Discharge well	128	88.3	31	57.4	< 0.001^c^
ADL partially dependant	10	6.9	9	16.7	
ADL fully dependant	1	0.7	4	7.4	
Death	6	4.1	10	18.5	
Discharge GCS					
Full GCS	134	92.4	36	66.7	< 0.001^c^
GCS 8 < X <15	5	3.5	4	7.4	
GCS < 8	6	4.1	14	25.9	
Post-op complication					
No	131	89.6	29	53.7	< 0.001^c^
Deep vein thrombosis	1	0.7	4	7.4	
Re-surgery	13	8.9	21	38.9	
Causes for re-surgery					
Haematoma	8	5.5	10	18.5	< 0.001^c^
Infarct	2	1.4	3	5.6	
Infected wound for wound debridement	2	1.4	8	14.8	
Hydrocephalus need shunt	1	0.7	0	0	
Follow-up					
3 months					
Mild disable (MRS 0–2)	136	93.8	37	68.5	< 0.001^c^
Moderate disable (MRS 3–4)	3	2.1	5	9.3	
Severe disable (MRS 5)	0	0	2	3.7	
Death	6	4.1	10	18.5	
6 months					
Mild disable (MRS 0–2)	136	93.8	37	68.5	< 0.001^c^
Moderate disable (MRS 3–4)	3	2.1	6	11.1	
Severe disable (MRS 5)	0	0	1	1.9	
Death	6	4.1	10	18.5	
12 months					
Mild disable (MRS 0–2)	137	94.5	37	68.5	< 0.001^c^
Moderate disable (MRS 3–4)	2	1.4	6	11.1	
Severe disable (MRS 5)	0	0	1	1.9	
Death	6	4.1	10	18.5	
18 Months					
Mild disable (MRS 0–2)	137	94.5	37	68.5	< 0.001^c^
Moderate disable (MRS 3–4)	2	1.4	5	9.3	
Severe disable (MRS 5)	0	0	0	0	
Death	6	4.1	12	22.2	
Overall Survival (OS) in months (mean, SD)	47.8 (18.2)		37.6 (20.94)		0.187^b^

a Chi-square test

b Independent *t*-test

c Fisher Exact test

**Table 4:** Univariate and multivariate Cox Regression on Predictors of Survival outcome

	Died (*n* = 28)	Alive (*n* = 171)	Univariable analysis			Multivariable analysis*		
			
	*n* (%)	*n* (%)	Crude HR	95% CI	*P*-value	Adj. HR	95% CI	*P*-value
Age Group								
< 40	2 (7.1)	16 (9.4)	1.00			1.00		
40–60	15 (53.6)	109 (63.7)	0.56	(0.124, 2.529)	0.451	1.58	(0.284, 8.716)	0.603
> 60	11 (39.3)	46 (26.9)	0.61	(0.280, 1.328)	0.213	1.98	(0.312, 12.551)	0.469
Gender								
Male	10 (35.7)	55 (32.2)	1.19	(0.549, 2.578)	0.66	1.63	(0.716, 3.742)	0.242
Female	18 (64.3)	116 (67.8)	1.00			1.00		
Ethnicity								
Malay	20 (71.4)	118 (69.0)	1.19	(0.521, 2.685)	0.689	1.19	(0.495, 2.842)	0.702
Non-Malay	8 (28.6)	53 (31.0)	1.00			1.00		
Comorbidities								
No	8 (28.6)	92 (53.8)	1.00			1.00		
Single	14 (50.0)	58 (33.9)	2.46	(1.033, 5.869)	0.042	1.83	(0.703, 4.760)	0.216
Multiple	6 (21.4)	21 (12.3)	3.00	(1.029, 8.549)	0.044	3.01	(0.827, 10.916)	0.095
Location of Meningiomas								
Skull base	18 (64.3)	36 (21.1)	5.49	(2.53, 11.90)	< 0.001	4.27	(1.504, 12.129)	0.006
Non-skull base	10 (35.7)	135 (78.9)	1.00			1.00		
WHO grading								
Grade 1	25 (89.3)	169 (98.8)	1.00			1.00		
Grade 2	3 (10.7)	2 (1.2)	5.89	(1.768, 19.616)	0.004	5.22	(1.256, 21.673)	0.023
Extents of Excision^a^								
Gross Total Resection (Simpsons 1–3)	11 (39.3)	142 (83.0)	1.00			1.00		
Subtotal Resection (Simpsons 4)	29 (17.0)	29 (17.0)	5.64	(2.641, 12.048)	< 0.001	1.96	(0.710, 5.408)	0.194

# Cox Regression, HR (hazard ratio)

* Adjusted with age, gender, ethnicity, comorbidities, location of tumour, WHO grading, extent of meningioma excision

a Extents of excision as quoted in corresponding EORTC/RTOG definitions as quoted in Jenkinson et al. (14)

**Table 4a:** Univariate Cox Regression on Predictors of Survival outcome

	Died (*n* = 28)	Alive (*n* = 171)	*P*-value^a^	Univariable analysis		
		
	*n* (%)	*n* (%)		Crude HR	95% CI	*P*-value
Age Group						
< 40	2 (7.1)	16 (9.4)		1.00		
40–60	15 (53.6)	109 (63.7)	0.403	0.56	(0.124, 2.529)	0.451
> 60	11 (39.3)	46 (26.9)	0.403	0.61	(0.280, 1.328)	0.213
Gender						
Male	10 (35.7)	55 (32.2)	0.710	1.19	(0.549, 2.578)	0.66
Female 18 (64.3)	116 (67.8)			1.00		
Ethnicity						
Malay	20 (71.4)	118 (69.0)	0.797	1.19	(0.521, 2.685)	0.689
Non-Malay	8 (28.6)	53 (31.0)		1.00		
Comorbidities						
No	8 (28.6)	92 (53.8)		1.00		
Single	14 (50.0)	58 (33.9)	0.044	2.46	(1.033, 5.869)	0.042
Multiple	6 (21.4)	21 (12.3)	0.044	3.00	(1.029, 8.549)	0.044
Location of Meningiomas						
Skull base	18(64.3)	36 (21.1)	< 0.001	5.49	(2.53, 11.90)	< 0.001
Non-skull base	10 (35.7)	135 (78.9)		1.00		
WHO grading						
Grade 1	25 (89.3)	169 (98.8)		1.00		
Grade 2	3 (10.7)	2 (1.2)	0.003	5.89	(1.768, 19.616)	0.004
Extents of Meningioma Excision						
Gross Total Resection (Simpsons 1–3)	1 (39.3)	142 (83.0)		1.00		
Subtotal Resection (Simpsons 4)	29 (17.0)	29 (17.0)	< 0.001	5.64	(2.641, 12.048)	< 0.001

a Chi-square test

# Cox Regression, HR: hazard ratio

CI: confidence interval

**Table 4b:** Multivariate Cox Regression on Predictors of Survival outcome

	Died (*n* = 28)	Alive (*n* = 171)	*P*-value^a^	Multivariable analysis*		
		
	*n* (%)	*n* (%)		Adjusted HR	95% CI	*P*-value
Age Group						
< 40	2 (7.1)	16 (9.4)		1.00		
40–60	15 (53.6)	109 (63.7)	0.403	1.58	(0.284, 8.716)	0.603
> 60	11 (39.3)	46 (26.9)	0.403	1.98	(0.312, 12.551)	0.469
Gender						
Male	10 (35.7)	55 (32.2)	0.710	1.63	(0.716, 3.742)	0.242
Female	18 (64.3)	116 (67.8)		1.00		
Ethnicity						
Malay	20 (71.4)	118 (69.0)	0.797	1.19	(0.495, 2.842)	0.702
Non-Malay	8 (28.6)	53 (31.0)		1.00		
Comorbidities						
No	8 (28.6)	92 (53.8)		1.00		
Single	14 (50.0)	58 (33.9)	0.044	1.83	(0.703, 4.760)	0.216
Multiple	6 (21.4)	21 (12.3)	0.044	3.01	(0.827, 10.916)	0.095
Location						
Skull base	18 (64.3)	36 (21.1)	< 0.001	4.27	(1.504, 12.129)	0.006
Non-skull base	10 (35.7)	135 (78.9)		1.00		
WHO grading						
Grade 1	25 (89.3)	169 (98.8)		1.00		
Grade 2	3 (10.7)	2 (1.2)	0.003	5.22	(1.256, 21.673)	0.023
Extents of Excision						
Gross Total (Simpsons 1–3)	11 (39.3)	142 (83.0)		1.00		
Subtotal (Simpsons 4)	29 (17.0)	29 (17.0)	< 0.001	1.96	(0.710, 5.408)	0.194

a Chi-square test

# Cox Regression, HR: Hazard Ratio

CI: Confidence Interval

* Adjusted with age, gender, ethnicity, comorbidities, location of tumour, WHO grading, extent of meningioma excision

**Table 5:** Multivariate Cox Regression analysis on survival outcome and skull-base meningioma with adjusted variable selection

	Died (*n* = 28)	Alive (*n* = 171)	Multivariable analysis*		
		
	*n* (%)	*n* (%)	Adjusted HR	95% CI	*P*-value
Skull Base	18 (64.3)	36 (21.1)	4.22	(1.53, 11.66)	0.005
Non-skull Base	10 (35.7)	135 (78.9)	1.00		

* Adjusted with gender, comorbidities, location of tumour, WHO grading and extent of meningioma excision

**Table 6:** Outcome after surgical resection of skull base meningiomas of different locations [modified after Chen et al. (7)]

Location	Rate of total excision (%)	Morbidity (%)	Mortality (%)
Anterior fossa	85–100	0–31.3	0–4.9
Tuberculum Sellae	76.4–93	25–45	0–8.7
Medial Sphenoid Ridge	58–87	5.7–13	0
Clinoidal	54.5–86.7	4–29	0
Middle fossa, Cavernous sinus	0	7.5–15	0
Posterior fossa, Petroclival	0–48	20.3–47	0–0.7
Cerebellopontine angle	82–86.1	10.4–35.7	0–5
Foramen Magnum	67–96	5.9–27	0–4.9
Jugular Foramen	50–100	30–61.5	0–20
Tentorial	77–91.3	9.7–55	0–3.7
